# Dietary Sea Buckthorn Polysaccharide Supplementation Enhances Antioxidant Capacity, Modulates Inflammatory Cytokines, and Alters the Serum Metabolome in Tumbler Pigeons

**DOI:** 10.3390/ani16172652

**Published:** 2026-08-24

**Authors:** Hui Chen, Qiming Zheng, Haiying Li, Chen Ma, Xiaobin Li, Xinsheng Guo, Kunyu Liao

**Affiliations:** College of Animal Science, Xinjiang Agricultural University, Urumqi 830052, China; m13201308339@163.com (H.C.); 17839750693@163.com (Q.Z.);

**Keywords:** sea buckthorn polysaccharide, tumbler pigeon, antioxidant capacity, inflammatory cytokines, serum metabolomics, purine metabolism, bile acid metabolism

## Abstract

Sea buckthorn polysaccharide (SP) has antioxidant and anti-inflammatory activities, but its effects in tumbler pigeons remain unclear. Eighty-four male tumbler pigeons were assigned to four treatments receiving diets containing 0, 100, 200, or 400 mg/kg effective SP for 60 days, with seven replicate cages per treatment. SP supplementation enhanced serum antioxidant capacity and modulated inflammatory cytokine profiles, with the most pronounced overall responses observed at 200 mg/kg. The SP200 and SP400 groups also had lower serum lactate, total cholesterol, and triglyceride concentrations than the control group, whereas most blood gas, electrolyte, and acid–base variables were unaffected. Because SP200 produced the strongest antioxidant and cytokine responses, the CON and SP200 groups were selected for untargeted serum metabolomics. This analysis identified 68 and 79 differential metabolites in positive- and negative-ion modes, respectively. KEGG enrichment analysis ranked purine metabolism, phenylalanine metabolism, primary bile acid biosynthesis, and the citrate cycle among the leading pathways; however, none remained significant after Benjamini–Hochberg correction. Exploratory Spearman analysis revealed associations between selected metabolites and phenotypic indicators. Taken together, these findings indicate that 200 mg/kg SP may be an effective dietary supplementation level for improving antioxidant and inflammatory status in tumbler pigeons, while the associated metabolomic changes warrant further targeted validation.

## Simple Summary

Tumbler pigeons are exposed to considerable physiological demands during routine flight and training, making nutritional strategies that support antioxidant and metabolic homeostasis of practical interest. This study evaluated the effects of dietary sea buckthorn polysaccharide supplementation on serum biochemical indices, antioxidant capacity, inflammatory cytokines, blood gas variables, and metabolomic profiles in flying tumbler pigeons. Supplementation, particularly at 200 mg/kg, enhanced antioxidant capacity, modulated inflammatory cytokine profiles, and reduced serum lactate and lipid concentrations without markedly affecting blood gas or acid–base status. Serum metabolomic analysis further identified coordinated changes in metabolites related to purine, bile acid, and aromatic amino acid metabolism, providing additional insight into the metabolic responses associated with sea buckthorn polysaccharide supplementation. These findings suggest that sea buckthorn polysaccharide may be a promising dietary supplement for supporting physiological homeostasis in tumbler pigeons.

## 1. Introduction

Pigeons (*Columba livia domestica*) are among the most widely reared poultry species worldwide and are valued for their multiple economic roles, including meat, egg, ornamental, and racing purposes [1]. Tumbler pigeons are a distinctive athletic breed of ornamental pigeons, also known as roller or tumbling pigeons, whose characteristic behavior is performing consecutive backward somersaults during high-altitude flight. Darwin described this tumbling behavior as a “strictly inherited instinct” with a specific genetic basis in On the Origin of Species [2]. In recent years, tumbler pigeon competitions and tumbling events have gradually emerged as popular activities that combine ornamental value with economic returns [3]. Flight duration, tumbling frequency, and movement continuity are the core criteria for judging athletic performance in such events, directly determining competition rankings and economic value. However, during prolonged training and high-intensity competition, tumbler pigeons commonly exhibit insufficient endurance, rapid fatigue onset, and slow post-exercise recovery, limiting their athletic performance and economic potential [4,5].

Flight is a high-intensity aerobic metabolic activity accompanied by a dramatic increase in oxygen consumption and the massive generation of reactive oxygen species (ROS) [6]. When ROS accumulation exceeds the scavenging capacity of the endogenous antioxidant defense system, lipid peroxidation, protein carbonylation, and DNA oxidative damage can occur, leading to the loss of muscle cell membrane integrity and impaired muscle contractile function [7]. Simultaneously, strenuous exercise can activate inflammatory signaling pathways such as nuclear factor-κB (NF-κB), promoting the release of pro-inflammatory cytokines such as interleukin-1β (IL-1β) and interleukin-6 (IL-6), which further exacerbate tissue damage and fatigue [8,9]. Therefore, enhancing endogenous antioxidant capacity, suppressing excessive inflammatory responses, and maintaining energy metabolic homeostasis through nutritional strategies represent a promising approach to alleviating exercise-induced fatigue and improving the athletic performance of tumbler pigeons [10].

Sea buckthorn (*Hippophae rhamnoides* L.) is a medicinal and edible deciduous shrub whose fruits and leaves are rich in various bioactive compounds, including polysaccharides, flavonoids, vitamin C, and tocopherols [11]. Sea buckthorn polysaccharide (SP) is a major functional component of sea buckthorn, mainly composed of monosaccharides such as rhamnose, galactose, glucose, arabinose, and uronic acids, with its precise structure and bioactivity varying according to raw material source and extraction process [12]. Previous studies have demonstrated that SP exhibits multiple biological activities. At the antioxidant level, it can directly scavenge DPPH and ABTS free radicals and up-regulate the activities of endogenous antioxidant enzymes such as superoxide dismutase (SOD), catalase (CAT), and glutathione peroxidase (GSH-Px), thereby reducing malondialdehyde (MDA) content [13]. At the anti-inflammatory and immunomodulatory level, SP can inhibit the expression of pro-inflammatory cytokines such as IL-1β and IL-6, while promoting the secretion of the anti-inflammatory cytokine interleukin-4 (IL-4) [14]. At the energy metabolism level, SP can regulate lipid metabolism, improve the balance between glycolysis and aerobic oxidation, and has demonstrated anti-fatigue effects in exhaustive exercise models [15]. Collectively, these findings indicate that SP can alleviate exercise-induced fatigue through the combined effects of antioxidant, anti-inflammatory, and metabolic regulation, showing application potential in the nutritional management of athletic animals.

However, the evidence described above has been derived primarily from in vitro experiments, rodent models, and production-performance-oriented poultry studies. It remains unclear whether SP can exert similar antioxidant and anti-inflammatory protective effects in athletic pigeons, and the appropriate supplementation dose is unknown. More importantly, the metabolic responses associated with SP supplementation have not been characterized in athletic pigeons under routine flying conditions.

Therefore, the present study was conducted using tumbler pigeons under regular flying conditions, with SP supplementation at doses of 100, 200, and 400 mg/kg. We systematically evaluated the effects of dietary SP on serum biochemical parameters, antioxidant enzyme activities, inflammatory cytokines, and whole-blood gas and electrolyte variables, and combined these measurements with untargeted serum metabolomics to characterize the physiological and metabolic responses associated with SP supplementation. This study aimed to provide a theoretical basis for the rational nutritional application of SP in tumbler pigeons.

## 2. Materials and Methods

### 2.1. Animal Ethics Statement

This study was reviewed and approved by the Institutional Animal Care and Use Ethics Committee of Xinjiang Agricultural University (Urumqi, China; protocol permit number: 2020024).

### 2.2. Experimental Animals and Design

A total of 84 healthy male tumbler pigeons, approximately 12 months of age and with similar body weight (initial BW: 415.10 ± 23.20 g), were randomly assigned to four treatments in a completely randomized design. Each treatment consisted of seven replicate cages with three pigeons per cage. The four treatments were a basal diet without sea buckthorn polysaccharide (CON) and the basal diet supplemented to provide 100 (SP100), 200 (SP200), or 400 mg/kg (SP400) effective polysaccharide. The supplementation levels refer to the effective polysaccharide content. Because the commercial sea buckthorn polysaccharide product contained 30% polysaccharide (Nanjing Zelang Biotechnology Co., Ltd., Nanjing, China), the amount of product incorporated into each treatment diet was adjusted accordingly. The required amount of the commercial product was thoroughly mixed into the basal diet to achieve the designated supplementation level and ensure uniform distribution. All pigeons were housed in one loft containing 28 independent cages evenly distributed among the four treatments. Stocking density, lighting (16 h light: 8 h dark), ventilation, sanitation, feeders, and drinkers were maintained consistently across treatments. Ambient temperature was kept at 18–24 °C with relative humidity of 50–70%. Each pigeon received 30 g of the corresponding experimental diet daily in two equal meals, offered from 09:00 to 11:00 and from 15:00 to 17:00. Drinking water was provided ad libitum throughout the experimental period. Pigeons were allowed to fly freely under routine farm management without additional forced flight training. The adaptation period lasted 7 days, followed by a 60-day experimental period. Each cage was considered an independent experimental unit. The ingredient composition and nutrient levels of the basal diet are presented in Table 1.

### 2.3. Sample Collection and Serum Preparation

On day 60, before the morning feeding, blood samples were collected from all three pigeons in each replicate cage via the wing vein. One portion of fresh whole blood was collected into lithium-heparinized tubes for immediate blood gas analysis. The remaining blood was collected into tubes without anticoagulant, allowed to clot at room temperature for 30 min, and centrifuged at 3000× *g* for 10 min at 4 °C. Equal volumes of serum from the three pigeons within each cage were pooled to generate one cage-level composite serum sample, yielding seven independent composite serum samples per treatment. The same cage-level composite serum samples were used for all serum biochemical, antioxidant, and cytokine analyses, as well as for the untargeted metabolomic analysis of the CON and SP200 groups. Aliquots of the composite serum samples were stored at −80 °C until analysis.

### 2.4. Determination of Serum Biochemical Indices

Serum concentrations of lactate (LAC), total protein (TP), albumin (ALB), globulin (GLB), blood urea nitrogen (BUN), glucose (GLU), total cholesterol (TC), triglycerides (TG), and activities of aspartate aminotransferase (AST), alanine aminotransferase (ALT), lactate dehydrogenase (LDH), creatine kinase (CK), and hexokinase (HK) were measured using commercial assay kits (Beijing Huaying Biotechnology Research Institute, Beijing, China) as previously described [16]. All assays were performed according to the manufacturer’s instructions.

### 2.5. Serum Antioxidant Indices and Cytokines

Serum activities of superoxide dismutase (SOD), catalase (CAT), glutathione peroxidase (GSH-Px), total antioxidant capacity (T-AOC), and concentrations of malondialdehyde (MDA), interleukin-1β (IL-1β), interleukin-6 (IL-6), and interleukin-4 (IL-4) were determined using commercial assay kits (Beijing Huaying Biotechnology Research Institute, Beijing, China) according to the methods reported previously [16] and the manufacturer’s protocols.

### 2.6. Whole-Blood Gas, Electrolyte, and Acid-Base Analysis

Fresh lithium-heparinized whole blood was gently mixed and immediately analyzed using an i-STAT 1 portable clinical analyzer (Abbott Point of Care Inc., Princeton, NJ, USA). CG8+ cartridges were used to measure pH, partial pressure of carbon dioxide (pCO_2_), partial pressure of oxygen (pO_2_), sodium (Na^+^), potassium (K^+^), ionized calcium (iCa^2+^), and packed cell volume (PCV). Hemoglobin (Hb), total carbon dioxide (TCO_2_), bicarbonate (HCO_3_^−^), base excess (BE), and oxygen saturation (sO_2_) were calculated by the analyzer. Chloride (Cl^−^) was determined using CHEM8+ cartridges. The three pigeons within each cage were analyzed separately, and the cage mean value was used as the observational unit.

### 2.7. Serum Untargeted Metabolomics

Based on the overall phenotypic responses across the four treatments, the CON and SP200 groups were selected for untargeted serum metabolomic comparison because the SP200 group showed the most pronounced overall antioxidant and cytokine responses. Accordingly, the metabolomic analysis was designed to characterize metabolic features associated with the phenotypically most responsive tested level rather than to evaluate dose-dependent metabolomic responses across all supplementation levels.

Metabolite extraction and liquid chromatography–tandem mass spectrometry (LC-MS/MS) analysis were performed following the general protocol described by Dunn et al. [9]. Briefly, a 100 μL aliquot of each composite serum sample was mixed with 400 μL of pre-cooled 80% methanol, vortexed, incubated on ice for 5 min, and centrifuged at 15,000× *g* for 20 min at 4 °C. The supernatant was diluted with mass spectrometry-grade water to a final methanol concentration of 53% and centrifuged again. The resulting supernatant was used for analysis. A quality control (QC) sample was prepared by mixing equal volumes of each experimental sample, and a blank sample (53% methanol) was processed identically.

Separation was carried out on a Hypersil Gold C18 column (100 mm × 2.1 mm, 1.9 μm; Thermo Fisher Scientific, Waltham, MA, USA) at 40 °C with a flow rate of 0.2 mL/min. Mobile phase A was 0.1% formic acid in water, and mobile phase B was methanol. Mass spectrometric detection was performed on a Q Exactive™ HF-X mass spectrometer (Thermo Fisher Scientific) operated in both positive and negative electrospray ionization modes. The scan range was *m*/*z* 100–1500. The key parameters were as follows: spray voltage, 3.5 kV; sheath gas flow rate, 35 psi; auxiliary gas flow rate, 10 L/min; capillary temperature, 320 °C; S-lens RF level, 60; auxiliary gas heater temperature, 350 °C. MS/MS spectra were acquired in data-dependent acquisition mode.

Raw data files were converted to mzXML format using ProteoWizard (version 3.0). Peak detection, alignment, and quantification were performed with the XCMS package (version 3.2), as described by Smith et al. [10]. Metabolites were identified by matching accurate mass and retention time against the mzCloud, HMDB, and KEGG databases. After removing background ions based on blank samples, the data were normalized to the total peak area of the QC sample. Metabolites with a coefficient of variation > 30% in QC samples were excluded from further analysis.

### 2.8. Statistical Analysis

All statistical analyses used the cage as the experimental unit (*n* = 7 cages per treatment). The cage-level phenotypic data are provided in Dataset S1. Composite serum samples were used for serum biochemical, antioxidant, cytokine, and metabolomic analyses, whereas cage means of the three individually measured pigeons were used for blood gas, electrolyte, and acid–base variables. Metabolomic analyses included only the CON and SP200 groups.

Data were analyzed using IBM SPSS Statistics 24.0 (IBM Corp., Armonk, NY, USA) and R 4.3.2. Results are presented as treatment means with pooled standard errors of the mean (SEM). Normality and homogeneity of variances were assessed using the Shapiro–Wilk test and Levene’s test, respectively. Treatment effects were evaluated by one-way analysis of variance. When the overall treatment effect was significant, treatment means were compared using Duncan’s multiple-range test. Within a row, means without a common superscript letter were considered significantly different.

Dose responses were analyzed using the actual effective supplementation levels of 0, 100, 200, and 400 mg/kg. To improve numerical scaling, dose was expressed in units of 100 mg/kg, giving D values of 0, 1, 2, and 4, and fitted as a continuous variable in a quadratic polynomial regression model:

Y_ij_ = β_0_ + β_1_D_i_ + β_2_D_i_^2^ + ε_ij_

where Y_ij_ is the observation for replicate j at dose i, D_i_ is the scaled sea buckthorn polysaccharide dose, β_1_ and β_2_ are the linear and quadratic regression coefficients, respectively, and ε_ij_ is the residual error. Linear *p* and Quadratic *p* represent the significance of the linear and quadratic terms, respectively. Because the dose levels were unequally spaced, default equally spaced orthogonal polynomial coefficients were not used. Statistical significance was declared at *p* < 0.05, and 0.05 ≤ *p* < 0.10 was considered a tendency.

For metabolomics data, multivariate analyses were conducted using the metaX package (version 1.4.16). Principal component analysis (PCA) and partial least squares discriminant analysis (PLS-DA) were performed after Pareto scaling, and variable importance in projection (VIP) values were derived from PLS-DA. Univariate analysis was based on *t*-tests, and fold changes (FC) were calculated. Differential metabolites were identified using VIP > 1, *p* < 0.05, and FC > 1.2 or < 0.833. Volcano plots were generated using the ggplot2 package (version 3.3.4) in R (version 4.3.2). KEGG pathway enrichment was assessed using Fisher’s exact test, with *p*-values adjusted by the Benjamini–Hochberg procedure. Pathways with q < 0.05 were considered significant, whereas top-ranked pathways with q ≥ 0.05 were interpreted as exploratory. Spearman’s rank correlation analysis was used to assess associations between representative differential metabolites and phenotypic indicators, and the results were visualized as a correlation heatmap.

## 3. Results

### 3.1. Serum Biochemical Indices

The effects of sea buckthorn polysaccharide supplementation on the serum biochemical indices of tumbler pigeons are presented in Table 2. Sea buckthorn polysaccharide supplementation affected serum lactate (LAC; overall *p* = 0.010), which decreased linearly with increasing supplementation levels (linear *p* = 0.040). The SP200 and SP400 groups had lower LAC concentrations than the CON group, and the SP400 group also had a lower concentration than the SP100 group (*p* < 0.05).

Serum total cholesterol (TC) and triglyceride (TG) concentrations were affected by supplementation (overall *p* = 0.002 for both). The SP200 and SP400 groups had lower TC and TG concentrations than the CON and SP100 groups (*p* < 0.05), whereas no difference was observed between the SP200 and SP400 groups (*p* > 0.05). TC and TG tended to decrease linearly with increasing sea buckthorn polysaccharide supplementation (linear *p* = 0.081 and 0.099, respectively).

Serum blood urea nitrogen (BUN) differed among treatments (overall *p* = 0.014) and exhibited a quadratic response to the supplementation level (quadratic *p* = 0.045). BUN was higher in the SP100 group than in the CON and SP400 groups (*p* < 0.05), whereas the SP200 group had an intermediate value.

The overall treatment effects on total protein (TP) and albumin (ALB) were not significant (overall *p* = 0.126 and 0.204, respectively). Nevertheless, the linear and quadratic dose terms for TP were significant (linear *p* = 0.032; quadratic *p* = 0.034), whereas ALB tended to exhibit a linear response (linear *p* = 0.069). The overall treatment effect on globulin (GLB) tended to be significant (overall *p* = 0.082). GLB exhibited a quadratic response (quadratic *p* = 0.045) and tended to exhibit a linear response (linear *p* = 0.083).

### 3.2. Serum Glucose Concentration and Enzyme Activities

The effects of sea buckthorn polysaccharide supplementation on serum glucose concentration and enzyme activities are presented in Table 3. Supplementation did not affect serum glucose (GLU), aspartate aminotransferase (AST), lactate dehydrogenase (LDH), or creatine kinase (CK), with overall *p* values of 0.106, 0.226, 0.220, and 0.334, respectively. The overall treatment effect on alanine aminotransferase (ALT) tended to be significant (overall *p* = 0.081), whereas neither the linear nor the quadratic dose term was significant (linear *p* = 0.148; quadratic *p* = 0.449). Serum hexokinase (HK) activity was affected by sea buckthorn polysaccharide supplementation and exhibited both linear and quadratic responses (overall, linear, and quadratic *p* < 0.001). HK activity was highest in the SP200 group, followed by the SP400, SP100, and CON groups, with significant differences among all four treatments (*p* < 0.05).

### 3.3. Serum Antioxidant Status and Cytokine Concentrations

The effects of sea buckthorn polysaccharide supplementation on serum antioxidant status and cytokine concentrations are presented in Table 4. All measured antioxidant indices and cytokines were affected by supplementation and exhibited significant linear and quadratic responses (overall, linear, and quadratic *p* < 0.001).

Compared with the CON group, all supplemented groups had higher serum SOD, CAT, GSH-Px, and T-AOC and lower MDA (*p* < 0.05). The SP200 group had the highest SOD, CAT, and T-AOC values and the lowest MDA concentration, whereas GSH-Px activity was highest in the SP200 and SP400 groups (*p* < 0.05).

Serum IL-1β and IL-6 concentrations were lower, whereas IL-4 was higher, in the supplemented groups than in the CON group (*p* < 0.05). Among the supplemented treatments, the SP200 group had the lowest IL-6 concentration and was among the groups with the lowest IL-1β and highest IL-4 concentrations (*p* < 0.05).

### 3.4. Whole-Blood Gas, Electrolyte, and Acid–Base Variables

The effects of sea buckthorn polysaccharide supplementation on whole-blood gas, electrolyte, and acid–base variables are presented in Table 5. Most blood gas, oxygenation, electrolyte, and acid–base variables were unaffected by supplementation (overall *p* > 0.05).

Treatment effects were observed for blood pH, Cl^−^, iCa^2+^, and BE (overall *p* = 0.031, 0.032, 0.036, and 0.001, respectively). The SP200 group had a higher pH than the SP400 group; the SP400 group had a lower Cl^−^ concentration than the other groups; the SP100 group had a higher iCa^2+^ concentration than the CON and SP200 groups; and BE was higher in the CON and SP200 groups than in the SP100 and SP400 groups (*p* < 0.05).

### 3.5. Serum Untargeted Metabolomics Analysis

#### 3.5.1. Data Quality and Multivariate Analysis

**Figure 1:**
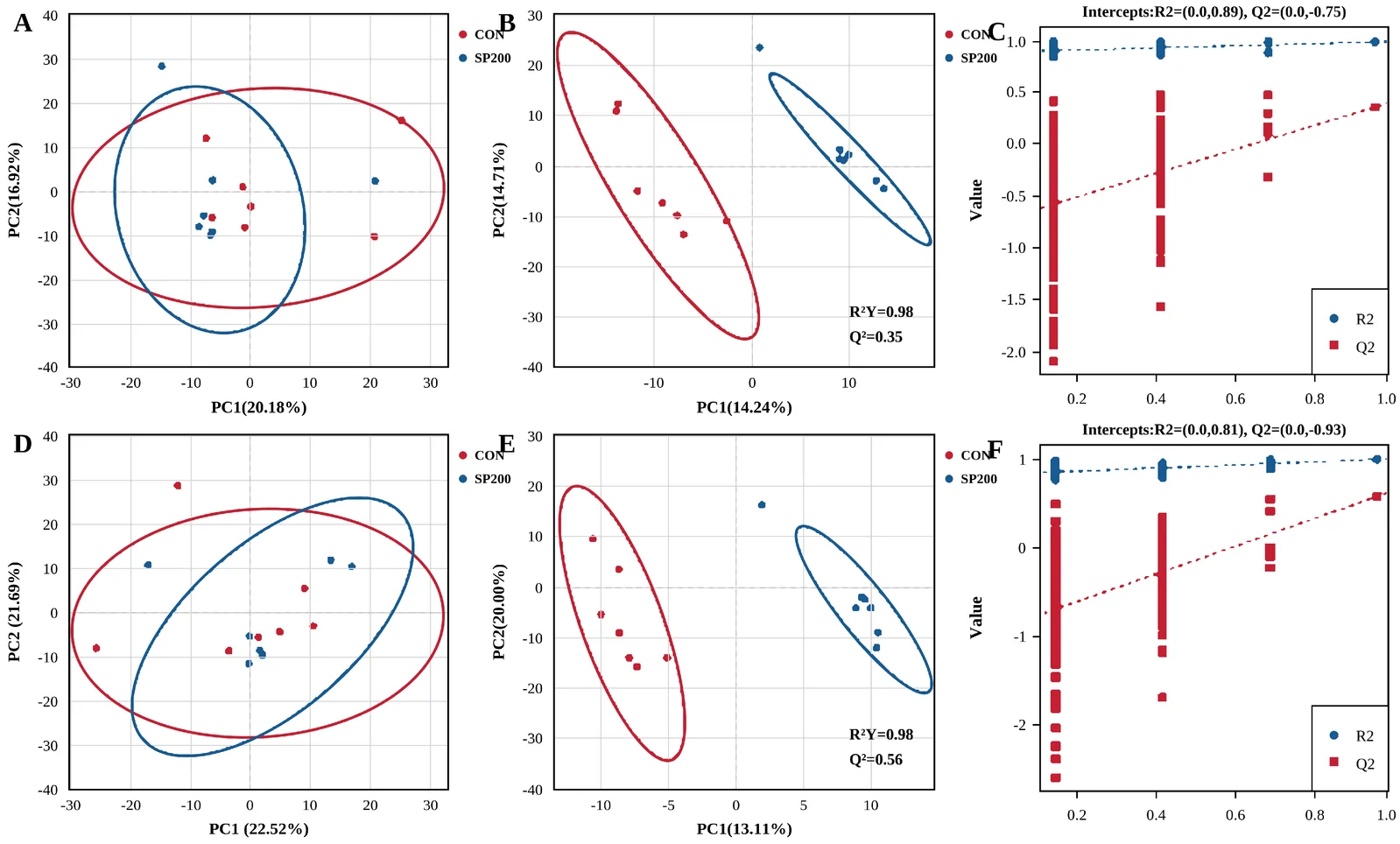
Multivariate analysis of serum metabolic profiles in the CON and SP200 groups. (**A**,**D**) PCA score plots, (**B**,**E**) PLS-DA score plots, and (**C**,**F**) permutation tests in the positive- and negative-ion modes, respectively (*n* = 7 cage-level samples per group). CON, control; SP200, 200 mg/kg effective sea buckthorn polysaccharide; PCA, principal component analysis; PLS-DA, partial least-squares discriminant analysis.

Untargeted serum metabolomics was performed in the CON and SP200 groups to characterize metabolic differences associated with the supplementation level showing the most pronounced overall phenotypic responses. The resulting serum metabolic profiles are presented in Figure 1. A total of 788 and 628 metabolic features were detected in the positive- and negative-ion modes, respectively. Pairwise correlation coefficients among the quality-control samples ranged from 0.995 to 0.996. In the negative-ion dataset, the median coefficient of variation was 7.6%, and 95.2% of the metabolic features had coefficients of variation below 30% (Figure S1). Principal component analysis showed partial separation between the CON and SP200 groups in both ionization modes (Figure 1A,D). The first two principal components explained 37.10% and 44.21% of the total variation in the positive- and negative-ion modes, respectively. Partial least-squares discriminant analysis separated the two groups in both ionization modes (Figure 1B,E). The positive-ion model had an R^2^Y of 0.98 and a Q^2^ of 0.35, whereas the negative-ion model had an R^2^Y of 0.98 and a Q^2^ of 0.56. The Q^2^ intercepts of the permutation tests were −0.75 and −0.93, respectively (Figure 1C,F).

#### 3.5.2. Differential Metabolite Analysis

**Figure 2:**
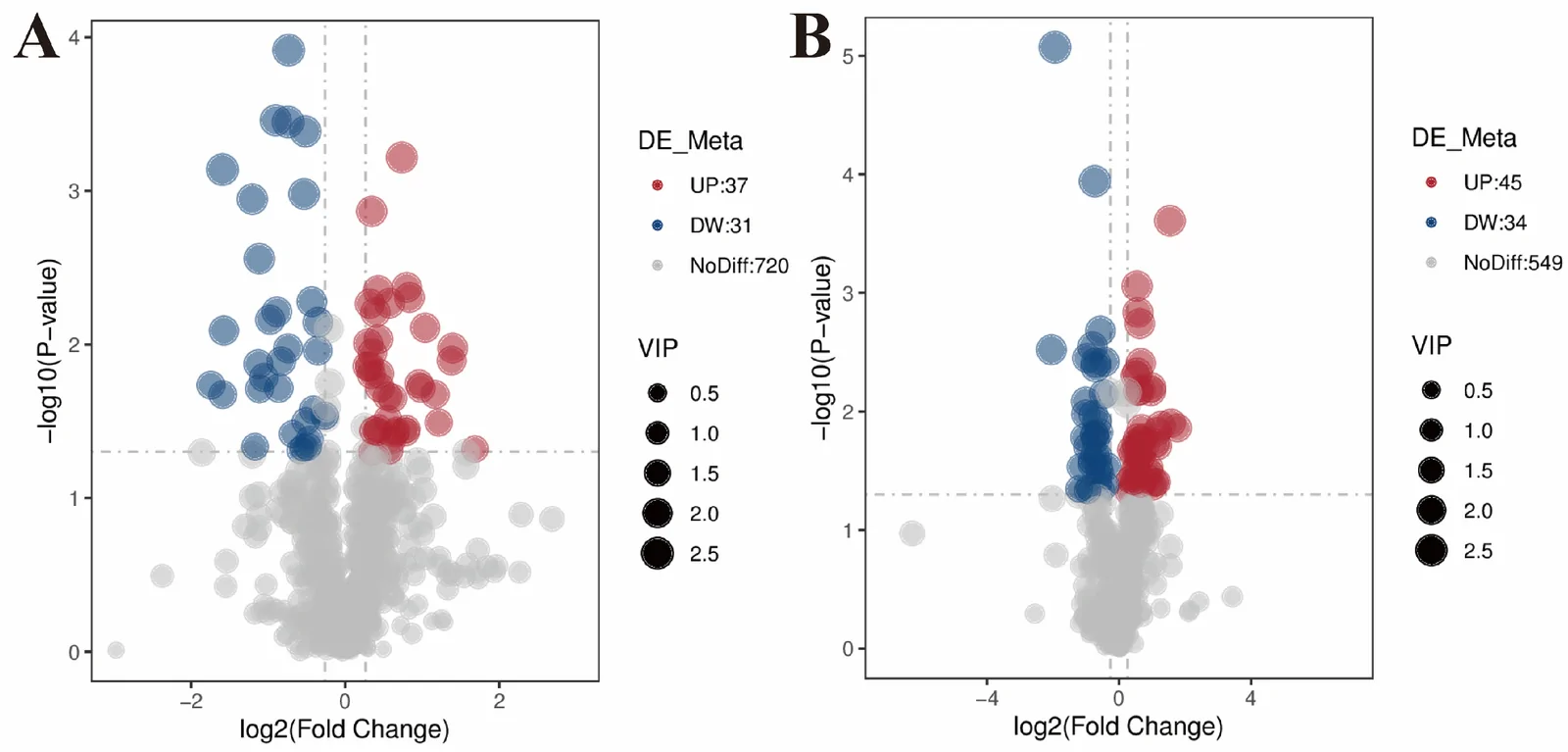
Volcano plots of differential serum metabolites between the SP200 and CON groups. (**A**) Positive-ion mode; (**B**) negative-ion mode. Red and blue indicate metabolites with higher and lower relative abundances in the SP200 group, respectively; gray indicates non-differential metabolites, and point size represents the VIP value. VIP, variable importance in projection. The dotted lines indicate the thresholds used for differential-metabolite screening: *p* < 0.05 and FC > 1.2 or < 0.833.

The differential metabolite screening results are presented in Figure 2. Based on the criteria of variable importance in projection > 1, *p* < 0.05, and a fold change >1.2 or <0.833, 68 differential metabolites were detected in the positive-ion mode, including 37 with higher and 31 with lower relative abundances in the SP200 group. In the negative-ion mode, 79 differential metabolites were detected, including 45 with higher and 34 with lower relative abundances in the SP200 group. The complete differential metabolite data for both ionization modes are provided in Dataset S2.

**Figure 3:**
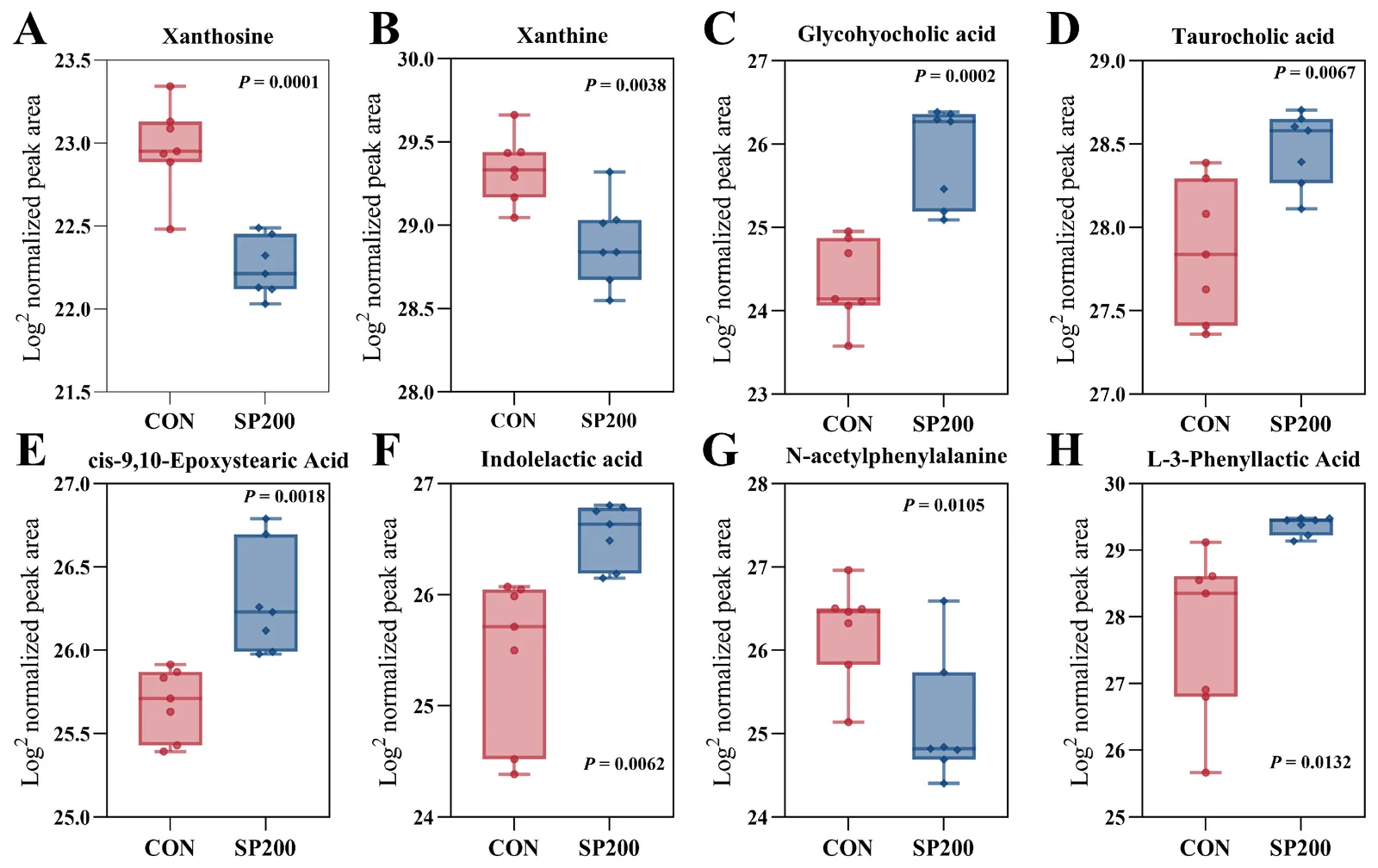
Relative abundances of eight representative serum metabolites in the CON and SP200 groups. Values are log_2_-transformed normalized peak areas (*n* = 7 cage-level samples per group). Displayed *p* values are from two-sided between-group comparisons.

The relative abundances of eight representative differential metabolites are presented in Figure 3. Compared with the CON group, the SP200 group had lower relative abundances of xanthosine (*p* < 0.001), xanthine (*p* = 0.0038), and N-acetylphenylalanine (*p* = 0.0105), but higher relative abundances of glycohyocholic acid (*p* < 0.001), taurocholic acid (*p* = 0.0067), cis-9,10-epoxystearic acid (*p* = 0.0018), indolelactic acid (*p* = 0.0062), and L-3-phenyllactic acid (*p* = 0.0132). Xanthosine remained significant after Benjamini–Hochberg correction (q < 0.05).

#### 3.5.3. Exploratory KEGG Pathway Enrichment Analysis

**Figure 4:**
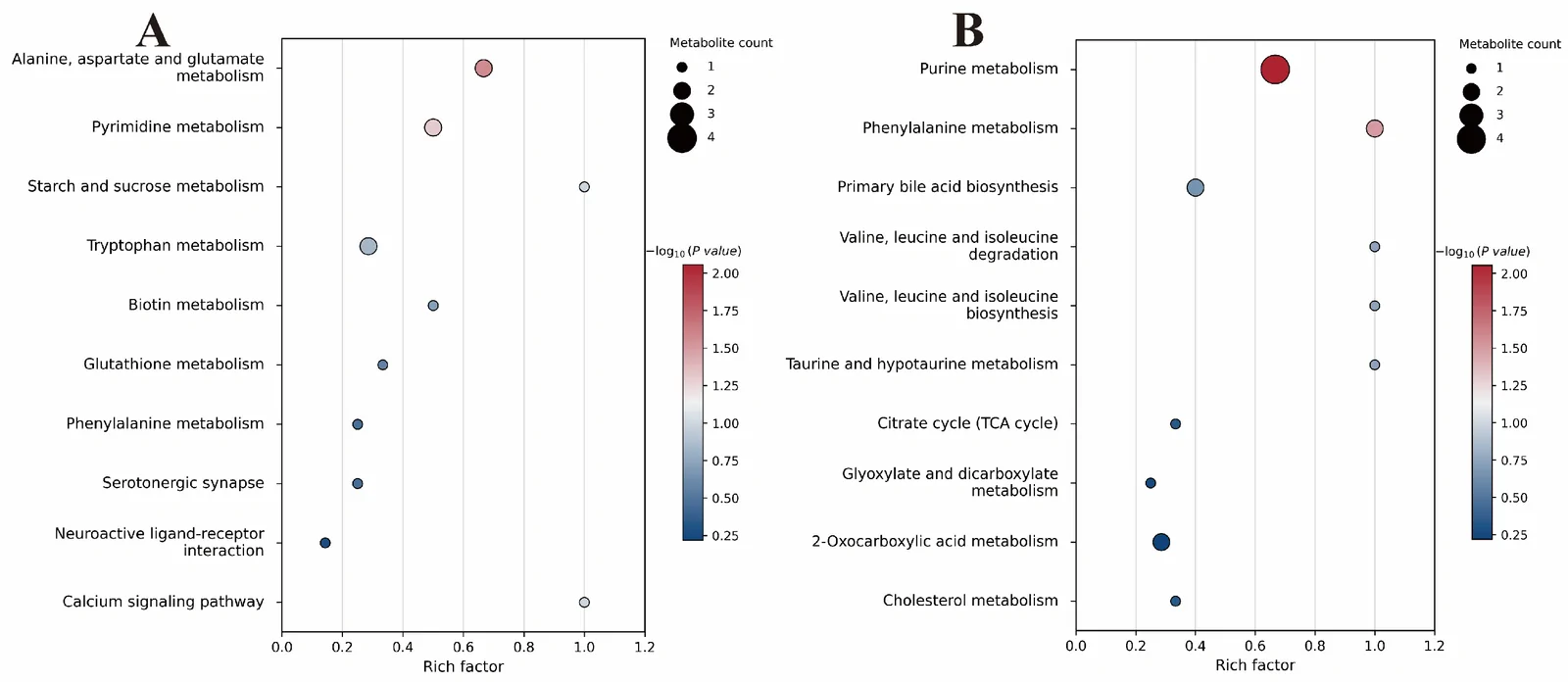
Exploratory KEGG pathway enrichment analysis of differential serum metabolites. The ten top-ranked pathways in the positive- (**A**) and negative-ion (**B**) modes are shown. The rich factor represents the proportion of annotated metabolites in a pathway that were identified as differential metabolites. Dot size indicates the number of differential metabolites mapped to each pathway, and dot color represents −log10(raw *p*). None of the pathways remained statistically significant after Benjamini–Hochberg correction (all q > 0.05). KEGG, Kyoto Encyclopedia of Genes and Genomes.

The KEGG pathway enrichment results are presented in Figure 4. In the positive-ion mode, the top-ranked pathways included alanine, aspartate and glutamate metabolism, tryptophan metabolism, glutathione metabolism, pyrimidine metabolism, and phenylalanine metabolism (Figure 4A). In the negative-ion mode, the top-ranked pathways included purine metabolism, phenylalanine metabolism, primary bile acid biosynthesis, taurine and hypotaurine metabolism, the citrate cycle, and cholesterol metabolism (Figure 4B). However, none of the pathways remained statistically significant after Benjamini–Hochberg correction (all q > 0.05); therefore, these pathway-level results were considered exploratory (Figure S2).

### 3.6. Correlations Between Representative Differential Metabolites and Phenotypic Indicators

**Figure 5:**
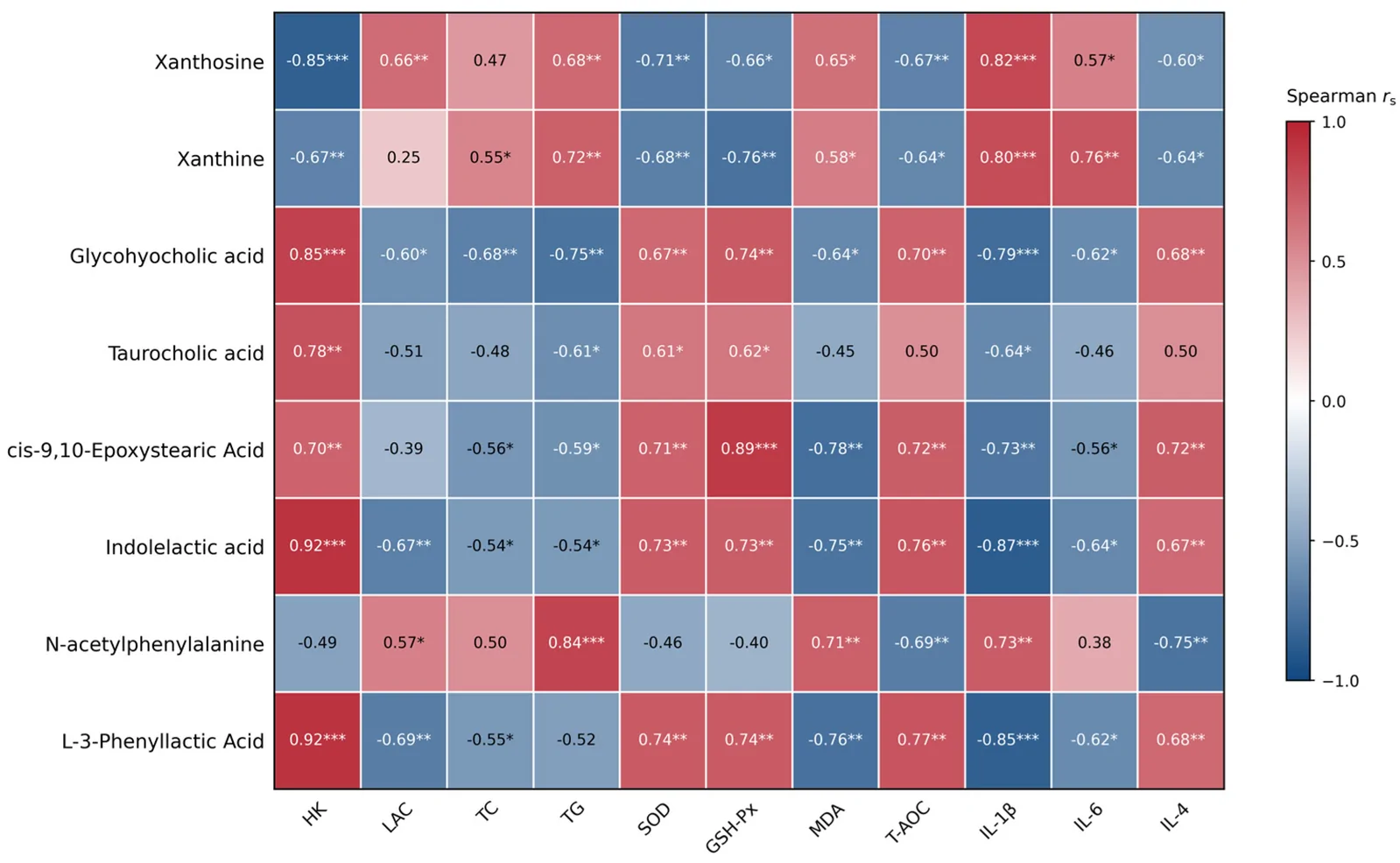
Spearman correlation heatmap between representative serum metabolites and phenotypic indicators. Correlations were calculated across 14 cage-level composite serum samples (*n* = 7 cages per group) from the CON and SP200 groups. Cell values indicate Spearman’s r_s_; red and blue indicate positive and negative correlations, respectively. * *p* < 0.05, ** *p* < 0.01, and *** *p* < 0.001 based on two-sided tests without adjustment for treatment. HK, hexokinase; LAC, lactate; TC, total cholesterol; TG, triglycerides; SOD, superoxide dismutase; GSH-Px, glutathione peroxidase; MDA, malondialdehyde; T-AOC, total antioxidant capacity; IL, interleukin.

Exploratory Spearman correlation analysis was performed across the 14 cage-level samples from the CON and SP200 groups to assess associations between representative differential metabolites and phenotypic indicators (Figure 5). Indolelactic acid and L-3-phenyllactic acid were positively correlated with HK (r_s_ = 0.921 and 0.916, respectively) and negatively correlated with IL-1β (r_s_ = −0.868 and −0.855, respectively; all *p* < 0.001). Cis-9,10-epoxystearic acid was positively correlated with GSH-Px (r_s_ = 0.890, *p* < 0.001), whereas xanthosine was negatively correlated with HK (r_s_ = −0.855, *p* < 0.001).

## 4. Discussion

### 4.1. SP Improved Antioxidant Capacity and Attenuated Pro-Inflammatory Cytokines

Dietary SP supplementation increased serum antioxidant enzyme activities and altered cytokine concentrations, with the greatest overall responses observed at 200 mg/kg (Table 4). Serum SOD, CAT, GSH-Px, and T-AOC were higher, and MDA was lower, in all SP-supplemented groups compared with the CON group. These findings are consistent with previous reports that SP can scavenge free radicals in vitro and increase antioxidant enzyme activities in vivo [12,17,18]. In broilers under oxidative stress, dietary SP reduced MDA and restored GSH-Px and SOD activities [13], and similar effects have been observed in rodents [16]. The glutathione metabolism pathway was among the top-ranked pathways in the positive-ion metabolomics data (Figure 4A), providing additional, albeit exploratory, support for altered glutathione-related metabolism. Serum IL-1β and IL-6 were lower, and IL-4 was higher, in the SP-supplemented groups, again with the strongest responses at 200 mg/kg. This cytokine profile has also been reported for SP in immunosuppressed mice [15]. Previous studies have shown that SP can inhibit NF-κB activation in LPS-stimulated RAW264.7 macrophages [19,20], while reactive oxygen species are recognized upstream activators of NF-κB [7]. In the present study, the concurrent enhancement of antioxidant defenses and reduction in IL-1β and IL-6 at 200 mg/kg is consistent with the close interaction between oxidative and inflammatory processes [8,21,22] and is compatible with a potential involvement of NF-κB-related signaling. However, NF-κB activity was not directly measured, and its specific role in the response to SP therefore remains to be verified. The generally greater responses at 200 than at 400 mg/kg indicate a nonlinear dose–response to SP supplementation. The basis for the attenuated response at the higher dose remains unclear. Under the present experimental conditions, 200 mg/kg produced the greatest overall antioxidant and cytokine response.

### 4.2. Serum Biochemical and Blood Gas Responses to SP Supplementation

Serum HK activity increased in SP-supplemented groups (Table 3). Because GLU concentration did not differ among treatments, the higher HK activity may reflect altered glucose phosphorylation-related metabolism. Serum HK activity does not directly represent tissue glucose uptake or glycolytic flux, and these were not measured. Serum LAC was lower in the SP200 and SP400 groups than in the CON group (Table 2). During flight, LAC accumulates when glycolytic ATP production exceeds mitochondrial oxidative capacity, contributing to muscle acidosis [23,24,25,26]. The reduction in LAC may indicate lower lactate accumulation, enhanced lactate clearance, or both.

Serum TC and TG were lower in the SP200 and SP400 groups than in the CON and SP100 groups (Table 2). Circulating lipid concentrations reflect the net balance among dietary absorption, hepatic synthesis, tissue uptake, and biliary excretion. The reductions in TC and TG therefore indicate altered lipid handling, although the relative contributions of these processes were not determined [27].

Most blood gas, electrolyte, and acid–base variables were unaffected by SP supplementation (Table 5). The isolated differences in pH, Cl^−^, iCa^2+^, and BE lacked a consistent dose-dependent pattern. These findings suggest that SP did not broadly alter systemic acid–base or electrolyte homeostasis under the free-flight conditions of this study [28].

### 4.3. Untargeted Metabolomic Profiling Revealed Exploratory Metabolic Shifts

Because the SP200 group showed the most pronounced overall phenotypic responses, untargeted serum metabolomics was performed to compare the CON and SP200 groups. Although several pathways ranked highly in the KEGG enrichment analysis, none remained statistically significant after Benjamini–Hochberg correction (all q > 0.05). Therefore, the pathway-level findings discussed below should be interpreted as exploratory patterns rather than evidence of statistically supported pathway alterations [29].

In the SP200 group, xanthosine and xanthine were less abundant than in the CON group (Figure 3). Both are intermediates of purine degradation, and oxidation of xanthine by xanthine oxidase can generate reactive oxygen species [30,31]. The concurrent decreases in xanthosine and xanthine, together with the lower serum MDA concentration observed in the SP200 group, may reflect a shift in purine catabolism associated with reduced oxidative burden [32]. Notably, xanthosine remained significant after Benjamini–Hochberg correction, further supporting this metabolite-level difference. The citrate cycle was also among the top-ranked pathways, raising the possibility that SP supplementation influenced central carbon metabolism. This interpretation is biologically consistent with the concurrent increase in HK activity and decrease in serum LAC observed in the SP200 group, although the present data do not establish the underlying metabolic flux.

Glycohyocholic acid and taurocholic acid were more abundant in the SP200 group, while primary bile acid biosynthesis was among the top-ranked pathways (Figure 3 and Figure 4B). Bile acids not only facilitate lipid absorption but also act as metabolic signaling molecules through nuclear and membrane receptors, including FXR and TGR5 [18,33,34]. Although these receptors were not examined in the present study, the parallel increase in circulating conjugated bile acids and decrease in serum TC and TG suggests a potential link between altered bile acid profiles and lipid handling following SP supplementation. Dietary polysaccharides have been reported to modulate bile acid metabolism, potentially through interactions with the gut microbiota [20,25,35,36]. Whether a similar gut microbiota–bile acid axis contributes to the responses observed in tumbler pigeons warrants further investigation.

Indolelactic acid and L-3-phenyllactic acid were more abundant, whereas N-acetylphenylalanine was less abundant, in the SP200 group (Figure 3). Indolelactic acid can be generated through microbial tryptophan metabolism and has been associated with AhR-related antioxidant and intestinal barrier-protective functions [37,38]. L-3-Phenyllactic acid can also be produced by certain lactic acid bacteria [22,26]. Although the origins of these circulating metabolites were not determined in the present study, their coordinated changes may reflect remodeling of aromatic amino acid-related metabolism following SP supplementation [27]. Although AhR signaling itself was not examined here, the reported antioxidant and immunomodulatory activities of indolelactic acid [37,38] provide a plausible biological link between the observed metabolomic changes and the antioxidant and cytokine responses in the SP200 group. Whether these metabolites directly contribute to the physiological responses to SP remains to be determined.

Spearman correlation analysis combining the CON and SP200 groups further showed that indolelactic acid and L-3-phenyllactic acid were strongly and positively correlated with HK and negatively correlated with IL-1β, whereas cis-9,10-epoxystearic acid was positively correlated with GSH-Px (Figure 5). Xanthosine showed an inverse association with HK. These coordinated associations reinforce the biological coherence between the metabolomic and phenotypic responses, particularly those related to energy metabolism, antioxidant capacity, and inflammatory status. Nevertheless, because the correlation analysis pooled samples from two treatment groups, these relationships should be regarded as associative rather than causal [28,36].

### 4.4. Study Limitations and Future Directions

Several limitations of this study should be acknowledged. First, untargeted metabolomics was restricted to the CON and SP200 groups, and metabolic profiles at 100 and 400 mg/kg were not characterized. Consequently, the metabolomic data characterize the metabolic responses associated with the phenotypically most responsive supplementation level but do not define dose-dependent metabolic trajectories across the full range of SP supplementation. Second, serum samples were pooled by cage, precluding evaluation of inter-individual variability [39], while the seven cage-level replicates per treatment may have limited the statistical power to detect smaller treatment effects. Third, flight activity was not standardized under a controlled exercise protocol, and individual flight exposure was not quantified; performance outcomes such as flight duration or tumbling frequency were also not recorded. Consequently, whether the observed biochemical and metabolomic responses translate into enhanced flight performance remains to be determined. Fourth, the signaling mechanisms discussed in relation to the observed antioxidant, inflammatory, and metabolomic responses were inferred from previous studies and were not directly measured in the present experiment. Fifth, none of the enriched metabolic pathways remained significant after correction for multiple testing, and all metabolomic interpretations should be regarded as hypothesis-generating. Sixth, the correlation analysis combined samples from the CON and SP200 groups; therefore, some associations may partly reflect treatment-related group separation rather than purely within-group biological covariation. More broadly, the present findings were obtained from male tumbler pigeons at a single experimental site over a 60-day period, without gut microbiota profiling or supplementation above 400 mg/kg; these factors should be considered when extrapolating the results to broader populations and supplementation conditions.

Despite these limitations, the present study provides consistent evidence that dietary SP, particularly at 200 mg/kg, improved the antioxidant and inflammatory status of tumbler pigeons under free-flight conditions. These phenotypic improvements were accompanied by changes in serum biochemical profiles and exploratory metabolomic features related to purine, aromatic amino acid, bile acid, and lipid metabolism. Taken together, the phenotypic dose–response patterns indicate that 200 mg/kg produced the most pronounced overall antioxidant and cytokine responses among the supplementation levels tested. Based on the phenotypic dose–response observed in the present study, 200 mg/kg SP can be considered a practical dietary supplementation level for tumbler pigeons. Future studies incorporating standardized exercise protocols, targeted metabolomics, and molecular analyses are required to validate the proposed metabolic associations and to determine whether the observed biochemical improvements translate into enhanced flight performance [40].

## 5. Conclusions

Dietary sea buckthorn polysaccharide supplementation enhanced serum antioxidant capacity, modulated inflammatory cytokine profiles, and altered selected biochemical indices in tumbler pigeons under free-flight conditions, while most blood gas, electrolyte, and acid–base variables remained unaffected. Among the supplementation levels tested, 200 mg/kg produced the most pronounced overall antioxidant and cytokine responses. Untargeted serum metabolomics comparing the CON and SP200 groups further identified distinct changes in selected metabolites, with exploratory pathway-level patterns involving purine, bile acid, aromatic amino acid, and lipid-related metabolism. Although none of the KEGG pathways remained significant after Benjamini–Hochberg correction, the coordinated metabolomic and phenotypic responses provide a basis for further investigation of the metabolic processes associated with SP supplementation. Collectively, these findings support the potential of 200 mg/kg SP as a nutritional supplementation level for tumbler pigeons under the conditions of the present study. However, controlled exercise trials, longer-term safety assessment, targeted metabolomic and molecular validation, and evaluation of practical feasibility are required before conclusions can be drawn regarding flight-performance benefits or breeder-level recommendations.

## Figures and Tables

**Table 1:** Ingredient composition and nutrient levels of the basal diet.

Feed Ingredient	Content (%)	Nutrient Leve ^2^	Content
Corn	65	Dry matter (DM), %	91.87
Soybean meal	20	Organic matter (OM), % of DM	96.24
Wheat middlings	5	Metabolizable energy (ME), MJ/kg	12.32
Cottonseed protein	5	Crude protein (CP), %	18.34
Soybean oil	1	Ether extract (EE), %	2.00
Limestone (0–2 mm)	1	Crude fiber (CF), %	3.05
Limestone (2–4 mm)	1	Calcium (Ca), %	0.94
Premix ^1^	2	Total phosphorus (P), %	0.58
Total	100	Lysine (Lys), %	0.86
		Methionine (Met), %	0.44

Note: ^1^ The premix provided the following per kilogram of diet: vitamin A, 5330 IU; vitamin D3, 1300 IU; vitamin E, 30 mg; vitamin K3, 3.10 mg; vitamin B1, 2.00 mg; vitamin B2, 7.00 mg; biotin, 0.10 mg; folic acid, 1.30 mg; niacin, 37 mg; calcium pantothenate, 2300 mg; lysine, 5400 mg; and methionine, 2300 mg. ^2^ DM, OM, ME, CP, EE, and CF were analyzed values; Ca, P, Lys, and Met were calculated values. The same basal diet formulation was used for all treatments. Sea buckthorn polysaccharide was added to the treatment diets to provide 100, 200, or 400 mg/kg effective polysaccharide, whereas the CON diet contained no supplemental sea buckthorn polysaccharide.

**Table 2:** Effects of dietary sea buckthorn polysaccharide supplementation on serum biochemical indices of tumbler pigeons.

Item	CON	SP100	SP200	SP400	SEM	Overall *p*	Linear *p*	Quadratic *p*
LAC, mmol/L	2.98 ^a^	2.73 ^ab^	2.19 ^bc^	2.11 ^c^	0.193	0.010	0.040	0.218
TP, g/L	28.73	25.56	26.06	27.41	0.982	0.126	0.032	0.034
ALB, g/L	18.06	16.95	16.44	16.61	0.566	0.204	0.069	0.152
GLB, g/L	10.67	8.61	9.62	10.80	0.643	0.082	0.083	0.045
TC, mmol/L	11.64 ^a^	11.78 ^a^	9.75 ^b^	9.78 ^b^	0.433	0.002	0.081	0.375
TG, mmol/L	3.02 ^a^	3.27 ^a^	1.92 ^b^	2.13 ^b^	0.255	0.002	0.099	0.351
BUN, mg/dL	4.15 ^b^	9.33 ^a^	5.56 ^ab^	3.14 ^b^	1.31	0.014	0.114	0.045

Note: Values are means (*n* = 7 cages per treatment); each replicate was a pooled serum sample from three pigeons. CON, basal diet; SP100, SP200, and SP400, 100, 200, and 400 mg/kg effective sea buckthorn polysaccharide, respectively. LAC, lactate; TP, total protein; ALB, albumin; GLB, globulin; TC, total cholesterol; TG, triglycerides; BUN, blood urea nitrogen; SEM, pooled standard error of the mean. Overall, linear, and quadratic *p* values represent the treatment effect and linear and quadratic dose responses, respectively. Means with different superscripts differ at *p* < 0.05.

**Table 3:** Effects of dietary sea buckthorn polysaccharide supplementation on serum glucose concentration and enzyme activities of tumbler pigeons.

Item	CON	SP100	SP200	SP400	SEM	Overall *p*	Linear *p*	Quadratic *p*
GLU, mmol/L	21.21	20.16	19.36	18.92	0.667	0.106	0.126	0.363
AST, U/L	248.38	231.52	185.26	176.49	28.05	0.226	0.259	0.541
ALT, U/L	38.92	33.80	29.95	26.66	3.31	0.081	0.148	0.449
LDH, U/L	143.58	94.79	93.47	83.36	21.47	0.220	0.129	0.274
CK, U/L	784.89	655.22	561.21	555.86	98.22	0.334	0.174	0.340
HK, U/mL	17.79 ^d^	24.88 ^c^	33.61 ^a^	27.85 ^b^	0.987	<0.001	<0.001	<0.001

Note: Values are means (*n* = 7 cages per treatment); each replicate was a pooled serum sample from three pigeons. CON, basal diet; SP100, SP200, and SP400, 100, 200, and 400 mg/kg effective sea buckthorn polysaccharide, respectively. GLU, glucose; AST, aspartate aminotransferase; ALT, alanine aminotransferase; LDH, lactate dehydrogenase; CK, creatine kinase; HK, hexokinase; SEM, pooled standard error of the mean. Overall, linear, and quadratic *p* values represent the treatment effect and linear and quadratic dose responses, respectively. Means with different superscripts differ at *p* < 0.05.

**Table 4:** Effects of dietary sea buckthorn polysaccharide supplementation on serum antioxidant status and cytokine concentrations of tumbler pigeons.

Item	CON	SP100	SP200	SP400	SEM	Overall *p*	Linear *p*	Quadratic *p*
SOD, U/mL	48.77 ^d^	59.12 ^c^	84.20 ^a^	72.83 ^b^	2.84	<0.001	<0.001	<0.001
CAT, U/mL	33.11 ^d^	37.62 ^c^	48.58 ^a^	43.70 ^b^	1.12	<0.001	<0.001	<0.001
GSH-Px, U/mL	119.26 ^c^	147.32 ^b^	179.31 ^a^	169.85 ^a^	3.97	<0.001	<0.001	<0.001
MDA, nmol/mL	4.132 ^a^	3.643 ^b^	2.560 ^d^	3.059 ^c^	0.124	<0.001	<0.001	<0.001
T-AOC, U/mL	5.714 ^d^	6.534 ^c^	9.006 ^a^	7.652 ^b^	0.192	<0.001	<0.001	<0.001
IL-1β, pg/mL	35.27 ^a^	26.73 ^b^	16.62 ^c^	18.77 ^c^	1.46	<0.001	<0.001	<0.001
IL-6, pg/mL	167.32 ^a^	150.14 ^b^	122.91 ^c^	139.92 ^b^	4.82	<0.001	<0.001	<0.001
IL-4, pg/mL	4.21 ^c^	5.57 ^b^	8.08 ^a^	6.97 ^a^	0.393	<0.001	<0.001	<0.001

Note: Values are means (*n* = 7 cages per treatment); each replicate was a pooled serum sample from three pigeons. CON, basal diet; SP100, SP200, and SP400, 100, 200, and 400 mg/kg effective sea buckthorn polysaccharide, respectively. SOD, superoxide dismutase; CAT, catalase; GSH-Px, glutathione peroxidase; MDA, malondialdehyde; T-AOC, total antioxidant capacity; IL, interleukin; SEM, pooled standard error of the mean. Overall, linear, and quadratic *p* values represent the treatment effect and linear and quadratic dose responses, respectively. Means with different superscripts differ at *p* < 0.05.

**Table 5:** Effects of dietary sea buckthorn polysaccharide supplementation on whole-blood gas, electrolyte, and acid-base variables of tumbler pigeons.

Item	CON	SP100	SP200	SP400	SEM	Overall *p*	Linear *p*	Quadratic *p*
pH	7.564 ^ab^	7.550 ^ab^	7.587 ^a^	7.532 ^b^	0.014	0.031	0.178	0.079
pCO_2_, mmHg	36.04	35.99	33.83	37.41	1.19	0.170	0.176	0.101
pO_2_, mmHg	40.80	38.44	46.00	40.10	2.44	0.121	0.199	0.194
Na^+^, mmol/L	151.42	154.81	146.63	150.09	2.38	0.092	0.311	0.423
K^+^, mmol/L	4.05	4.18	4.22	4.24	0.087	0.504	0.280	0.434
Cl^−^, mmol/L	131.02 ^a^	130.47 ^a^	129.68 ^a^	120.03 ^b^	3.09	0.032	0.753	0.272
iCa^2+^, mmol/L	1.16 ^b^	1.30 ^a^	1.15 ^b^	1.22 ^ab^	0.039	0.036	0.979	0.956
PCV, %	57.80	56.56	58.22	58.90	1.15	0.451	0.966	0.711
TCO_2_, mmol/L	33.62	32.68	32.10	32.50	0.966	0.786	0.332	0.394
Hb, g/dL	19.66	19.20	19.83	20.02	0.404	0.444	0.997	0.756
HCO_3_^−^, mmol/L	32.56	31.59	31.12	31.37	0.940	0.790	0.352	0.428
BE, mmol/L	5.83 ^a^	4.53 ^b^	5.72 ^a^	3.83 ^b^	0.384	0.001	0.610	0.214
sO_2_, %	83.19	79.73	85.80	80.39	2.26	0.173	0.439	0.362

Note: Values are means (*n* = 7 cages per treatment); each replicate was the mean of three pigeons housed in the same cage. CON, basal diet; SP100, SP200, and SP400, 100, 200, and 400 mg/kg effective sea buckthorn polysaccharide, respectively. pCO_2_, partial pressure of carbon dioxide; pO_2_, partial pressure of oxygen; iCa^2+^, ionized calcium; PCV, packed cell volume; TCO_2_, total carbon dioxide; Hb, hemoglobin; HCO_3_^−^, bicarbonate; BE, base excess; sO_2_, oxygen saturation; SEM, pooled standard error of the mean. Overall *p*, treatment effect; linear and quadratic *p*, linear and quadratic dose responses. Means with different superscripts differ at *p* < 0.05.

## Data Availability

The data that support the findings of this study are available from the corresponding author upon reasonable request.

## Supplementary Materials

The following supporting information can be downloaded at: https://www.mdpi.com/article/10.3390/ani16172652/s1, Figure S1: Quality-control assessment of the untargeted serum metabolomics data; Figure S2: Enrichment bubble diagram of complete KEGG pathway; Dataset S1: Cage-level phenotypic data of tumbler pigeons; Dataset S2: Complete differential serum metabolite data between the CON and SP200 groups in positive- and negative-ion modes.
