# Supplementary material for: Dietary Sea Buckthorn Polysaccharide Supplementation Enhances Antioxidant Capacity, Modulates Inflammatory Cytokines, and Alters the Serum Metabolome in Tumbler Pigeons
Source: Animals (Basel). 2026 Aug 24;16(17):2652. doi: 10.3390/ani16172652 (PMC13564298; doi:10.3390/ani16172652)
Supplement: Supplementary file 1 [file animals-16-02652-s001.zip › Supplementary Materials.pdf]

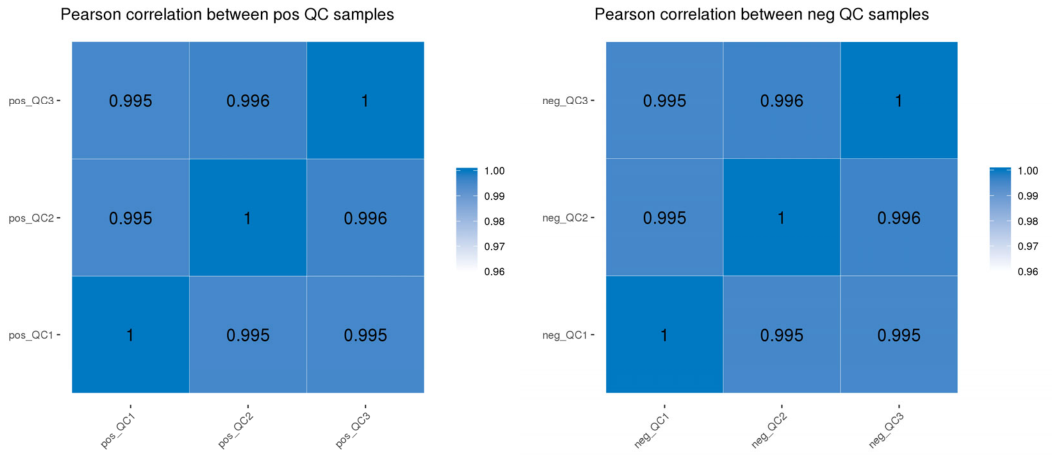

Figure S1. Quality-control assessment of the untargeted serum metabolomics data.

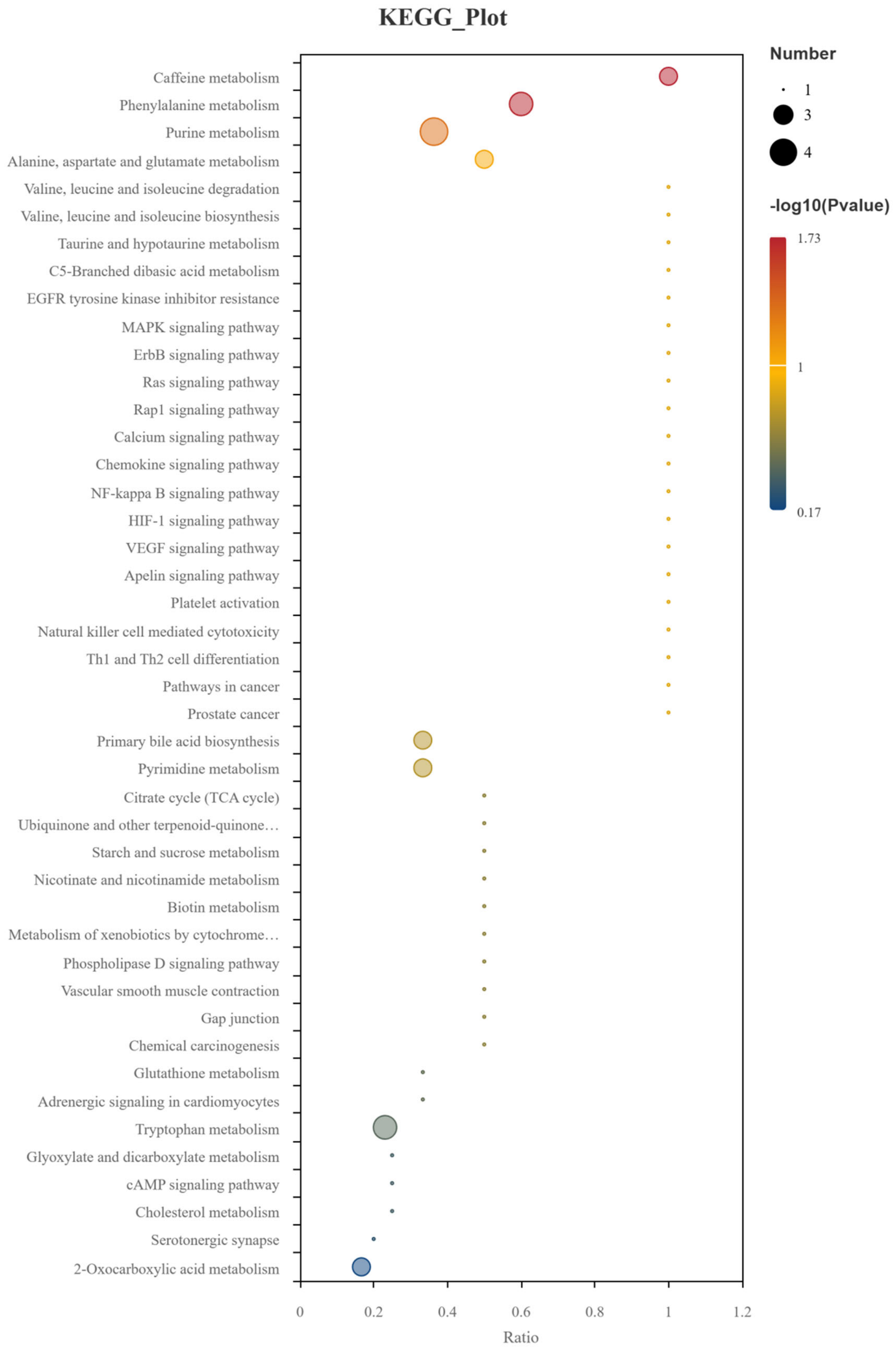

Figure S2.Enrichment bubble diagram of complete KEGG pathway
